# Effects of Integrative Cognitive Function Improvement Program on Cognitive Function, Oral Health, and Mental Health in Older People: A Randomized Clinical Trial

**DOI:** 10.3390/ijerph192114339

**Published:** 2022-11-02

**Authors:** Eun-Seo Jung, Yoon-Young Choi, Kyeong-Hee Lee

**Affiliations:** 1 Department of Dental Hygiene, College of Bioecological Health, Shinhan University, Uijeongbu 11644, Korea; 2 Private Practice, Nohyeong 14-gil, Jeju 63083, Korea

**Keywords:** cognition, cerebral blood flow, electroencephalogram, mental health, older adults, oral health

## Abstract

We aimed to investigate the effects of an integrative cognitive function improvement program that combined existing cognitive, emotional, and physical therapies on cognitive function, oral health, and mental health in elder participants. Participants were classified into one of the following groups: cognitively normal (CN; *n* = 18), mild cognitive impairment (MCI; *n* = 17), and control (*n* = 17). An integrative cognitive function improvement program was administered to the CN and MCI groups for six weeks. To measure cognitive function, electroencephalogram (EEG) and cerebral blood flow (CBF) were evaluated, and to measure oral health, the O’Leary index, Löe & Silness index, tongue coating, unstimulated saliva flow rate, and oral muscle strength were measured. To measure mental health status, mental health, happiness and social support were measured. The CN and MCI groups showed a significant change in EEG-based indices for awareness level and physical stress. Regarding oral health, the O’Leary and the Löe & Silness index score decreased significantly in the CN and MCI groups. Saliva flow rate increased significantly in the CN and MCI groups. In regards to mental health, the happiness score increased post-intervention in the CN and MCI groups. In conclusion, the integrative cognitive function improvement program was effective in improving cognitive function, oral health, and mental health of elder people.

## 1. Introduction

In general, the elder population can perform cognitive activities at the same level as younger people, as long as they do not have serious illness. However, brain function deteriorates and memory decreases gradually with increasing age. Approximately 25% of people between the ages of 60 and 69 show mild cognitive impairment and 54.6% of people ages 85 and above show moderate cognitive impairment [1,2]. Dementia is one representative disorder closely linked to cognitive function. In 2019, approximately 790,000 people living in Korea aged 65 and above were estimated to have dementia, and the number of cases is expected to increase to 3 million by 2050 [1]. To slow the progression of dementia or mitigate the disorder, pharmacological and non-pharmacological approaches have been suggested. The latter approach includes cognitive therapy, emotional therapy, and physical therapy [3].

Cognitive therapy is closely related to psychological, social, and physical functioning, and its focus is to improve various cognitive functions such as attention, computation, memory, language, and visuospatial discrimination [4]. In addition, oral health is reported to be strongly associated with cognitive function [5]. It was reported in some experiments conducted with mice that mastication could have a direct impact on cognitive function by increasing the number of cells produced in the dentate gyrus of the hippocampus and that loss of teeth modified the animals’ dietary pattern, eventually causing cognitive impairment [6,7]. Additionally, it was reported that tooth loss was a risk factor for the rapid progression of cognitive impairment and the occurrence of dementia, mastication was associated with an increase in cerebral blood flow (CBF), and fixed prosthetics and denture use enhanced cerebral function and CBF [8]. Therefore, oral health education is expected to positively impact cognitive function.

Emotional therapy can offer a positive change in the quality of life and vitalizes elder people faced with perceptual or physical function loss [9]. Depression is the most common mental health problem in old age and is linked to the worsening of cognitive function and the occurrence of dementia [10]. Of all the emotional therapy approaches, music therapy and laughter therapy are cost-effective compared to other interventions and reflect social aspects, as well. Hence, these therapies are recognized as being useful in motivating elder people experiencing loss and loneliness and in promoting mental health (e.g., improving depression, anxiety, and dissatisfaction with daily life) [11,12].

Physical therapy prevents cognitive impairment due to frontal lobe degeneration by improving cardiovascular function through physical activity and increasing CBF [13]. Of various physical activities, whole-body exercise increases cortical activity, positively influences cognition and cerebral function like memory and learning, and enhances the cognitive competence accountable for memory. Moreover, it is reported to be effective in preventing a decrease in CBF due to aging [14,15]. Mouth exercises are reported to be effective in strengthening oral muscles and increasing saliva production [16]. One study reported that oral muscle strength and saliva production increased more in the group which performed oral exercises in addition to whole-body exercises when compared to the group which performed oral exercises alone [17]. 

Individual differences exist in cognitive impairment in old age, and they manifest themselves in conjunction with other complex factors. Hence, compared to stand-alone therapies, an integrative program was found to be more effective in helping individuals with diverse symptoms (cognitive, emotional, and physical) by increasing participants’ interest and promoting active participation [18,19]. However, most previous studies in which the effect on cognitive function was investigated administered a single type of intervention to compare experimental and control groups and used the Mini-Mental Status Examination (MMSE) to assess cognitive function [20]. Though MMSE is appropriate in assessing the current level of cognitive function, it is difficult to scientifically prove cognitive improvement with this tool. Thus, it is necessary to conduct a study in which the effect of cognitive improvement is objectively measured.

Accordingly, this study was conducted to develop an integrative program composed of cognitive, emotional, and physical components to improve cognitive function in cognitively normal and mildly impaired elder participants and examine this program’s effects on cognitive function, oral health, and mental health.

## 2. Materials and Methods

### 2.1. Participants

Study participants were people aged 65 or higher who were members of a senior center in the Gyeonggi Province. The inclusion criteria for the participants included the following: (1) understanding of study purposes and voluntary consent for participation, (2) no problem in communicating with others, (3) being ambulatory, (4) undergoing no pharmacological therapy to improve cognitive function, and (5) no history of participating in other cognitive intervention programs. The minimal sample size required to test the mean differences was estimated using G * Power 3.1.9.2 for Windows (Heinrich-Heine-University Düsseldorf, Düsseldorf, Germany) and estimated to be *n* = 48 under the assumptions of a significance level of 0.05, an effect size of 0.25, and a power of 0.85. In this study, a total of 60 participants were enrolled in anticipation of drop-outs [21]. 

**Figure 1:**
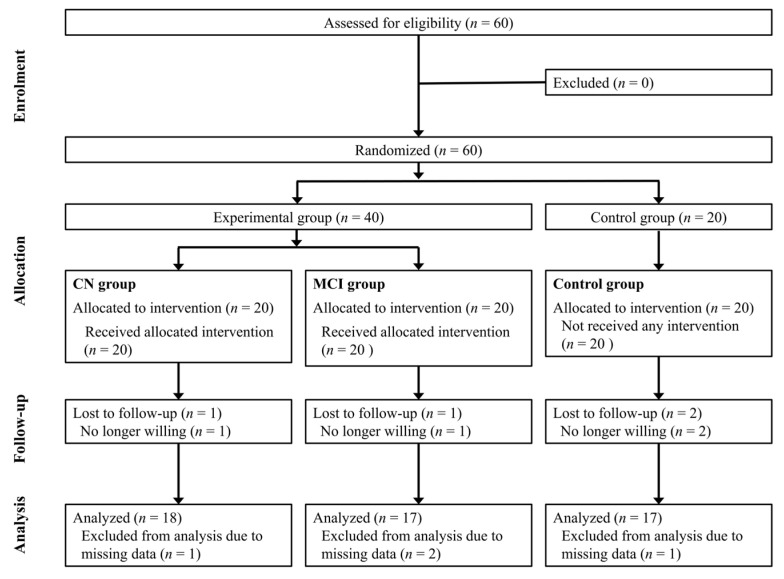
Consolidated Standards of Reporting Trials (CONSORT) diagram of participants in the randomized clinical trial. CN, cognitively normal; MCI, mild cognitive impairment.

Participants were randomly allocated to control (*n* = 20) and experimental groups (*n* = 40). The Korean version of the Mini-Mental Status Examination (MMSE-K) was used to classify participants in the experimental group into cognitively normal (CN) and mildly cognitively impaired (MCI) groups. Participants with a score of 24 or higher were classified into the CN group and those with a score between 18 and 23 were classified into the MCI group [20]. Data from a total of 52 participants (18 in the CN group and 17 each in the MCI and control groups) were submitted for analysis, after excluding those who withdrew themselves from the study and those who were lost to follow-up (Figure 1). For CBF measurements, four participants in the CN group were randomly selected.

### 2.2. Integrative Cognitive Function Improvement Program

The integrative cognitive function improvement program for the elder participants was developed in several phases. In the first phase, the literature was reviewed—studies in which cognitive function improvement programs were administered to elders in general or elders with mild cognitive impairment in the last 10 years were searched, and the relevant data were collected. In the second phase, program contents (cognitive, emotional, and physical activities) were determined based on the literature review. In the third phase, an expert group consisting of a dentist, two dental hygienists, and three professors of dental hygiene were consulted to evaluate the program’s adequacy. Based on their recommendations, parts of the program were revised. In the fourth phase, a pilot study was conducted with five people aged 65 or older who were members of a senior center in Uigeongbu City to estimate the length of time needed to administer the survey and intervention, perform a dental exam, and review the results. The pilot study revealed that the survey and dental exam were not time effective. Thus, they were not included in the time allocated to administer the program and instead, pre- and post-intervention tests were planned before the program began and after it was completed. In the fifth phase, based on the pilot study, the second round of revisions was made to finalize the program. The finalized program was designed as an integrative program consisting of cognitive, emotional, and physical activities in a group learning setting.

#### 2.2.1. Cognitive Activities: Oral Health Education and the Use of a Workbook

The cognitive activities consisted of oral health education and a post-education workbook developed in a previous study [22]. For oral health education, oral disease and the prevention of oral disease were discussed for 30 min per session. Additionally, the workbook was used in each session to review the lessons taught during the oral health education portion.

#### 2.2.2. Emotional Activities: Music Therapy and Laughter Therapy

Emotional activities consisted of music and laughter activities, performed for 30 min per session. In music therapy, familiar, simple, and repetitive songs have been used in previous studies to stimulate long- and short-term memories and to maintain focus and attention [11]. Laughter therapy consisted of greeting laughter, laughter in daily life, laughter exercise, clapping and laughing, body laughter, and singing laughter [12]. In consideration of the elder participants’ physical limitations, the laughter therapy was composed of activities that did not require significant physical energy and also allowed participants to take several breaks throughout the therapy session.

#### 2.2.3. Physical Activities: Whole-Body Exercise and Mouth Exercise

Physical activities included whole-body exercise to stimulate the cranial nerves and cognitive function by using the hand and facial muscles and mouth exercise to promote oral health. Participants performed the exercises for 30 min per session [17]. The physical activities were modified to be at an appropriate intensity level and rate, given the physical limitations of the elder participants. To prevent falls, the exercises were designed to be performed sitting in a chair. Additionally, the exercises were designed to be easy and repetitive so that the participants could perform them solo (Supplementary Table S1).

### 2.3. Interventions

Integrative cognitive function improvement program was administered to participants in the experimental groups in May and June 2022. In consideration of the executability and the duration for participants to maintain focus, two 90-min sessions were planned per week, for a total of six weeks. The participants learned the recommended cognitive, emotional, and physical activities by watching the researcher and instructor team repetitively demonstrate each of the activities. The control group did not undergo the integrative cognitive function improvement program during the study period. For ethical reasons, however, the program was administered for six weeks to the control group upon completion of the study in June 2022.

### 2.4. Research Tools

A week before the intervention began, the researcher and a trained research assistant administered a pre-intervention test to participants in all three groups over two days (2 and 3 May 2022). Participants filled out a self-report survey, received a dental exam, and underwent an electroencephalogram (EEG) study performed by an expert. A post-intervention test (self-report survey, dental exam, and EEG study) was performed using the same criteria a week after completion of the program. The EEG measurements were recorded a total of three times (pre-intervention, after the first session, and post-intervention). Four participants in the CN group visited a designated hospital to participate in a CBF study a total of three times (pre-intervention, after the first session, and post-intervention).

#### 2.4.1. Group Homogeneity Test

To test group homogeneity, data regarding demographics, cognitive function, activities of daily living (ADL), and quality of life with respect to oral health were collected by administering a self-report survey. To assess cognitive function, the MMSE-K was used [20]. Additionally, the Barthel Index was used to measure performance in ADL [23]. Oral health-related quality of life was assessed by using the Oral Health Impact Profile (OHIP-14) [24] and Geriatric Oral Health Assessment Index (GOHAI) [25]. Details of these indexes are shown in Supplementary Table S2.

#### 2.4.2. Outcome Measures

To examine cognitive function, EEG and CBF studies were conducted. EEG was measured with Neuro brain (Neuro21, Seoul, Korea), an EEG device developed by the Korea Institute of Brain Science [26]. Two electrodes were used to simultaneously measure brain waves in the prefrontal lobe, and the average values computed over the left and right measurements were recorded. In the study, brain waves in various frequency bands, namely, the delta (δ), theta (θ), alpha (α), sensorimotor rhythm (SMR), and beta (β) waves, were identified. The Korea Institute of Brain Science developed Brain Function Measurement (BFM) to quantify the recorded brain waves in an easy-to-understand manner [26]. In this study, the following indices were investigated using BFM: arousal level (AL), obtained by dividing the amplitude of the theta (θ) wave by that of the SMR wave and indicative of the level of resistance to extrinsic disease and stress; physical tension (PT), based on the amplitude of the delta (δ) wave and indicative of muscular or mental tension; mental distraction (MD), assessed by a high amplitude of the beta (β) wave and indicative of anxiety, tension, or excitation. An EEG study was conducted prior to the administration of the program, after the first session, and immediately after completion of the program, for a total of three times. To control inter-rater reliability, all of the EEG measurements were performed by a single researcher. Lower AL, PT, and MD values indicated a greater improvement in cognitive function.

CBF measures the volume of blood flow to the brain and is an indirect method to evaluate cognitive improvement. In this study, after consultation with experts, transcranial Doppler ultrasound (TCD) was selected from several CBF measurement approaches because it was relatively affordable, and the participants were more accepting of the approach [27]. The participants lay in a supine position on a portable massage bed, and a 2-MHz transducer connected to the TCD system was placed to measure blood flowing toward the area at a depth of 58 mm. The velocities averaged over the left and right CBF in the middle cerebral artery (MCA), anterior cerebral artery (ACA), posterior cerebral artery (PCA), vertebral artery, (VA), and basilar artery (BA) were recorded. Higher values indicated increased blood flow.

To examine oral health status, the O’Leary index (dental plaque) [28], Löe & Silness index (gingivitis) [29], tongue coating [30], unstimulated saliva flow rate [17], and oral muscle strength [31] were measured. Oral muscle strength was assessed by using the Iowa Oral Performance Instrument (IOPI) (IOPI Medical, Woodinville, WA, USA), a tool to derive objective data by measuring the pressure of anatomical structures in the mouth [31]. Oral muscle strength was measured in three areas: anterior tongue, posterior tongue, and cheek muscle strength (Supplementary Table S3).

To evaluate mental health status, mental health in old age, happiness in old age, and social support were assessed. Mental health in old age was assessed with the Korean General Health Questionnaire (KGHQ), which was developed based on Goldberg’s General Health Questionnaire [32]. Happiness in old age was assessed with a subjective happiness scale [33]. Social support was assessed by using the Medical Outcomes Study Social Support Survey (MOSSSS) developed by the Rand and Medical Outcomes Study team (Supplementary Tables S4 and S5) [34].

### 2.5. Data Analysis

The data were analyzed using IBM SPSS Statistics (version 22.0, IBM Corporation, New York, NY, USA). Statistical significance was determined if a *p* value < 0.05. To compare participant groups on demographic characteristics, the Chi-square test, Fisher’s exact test, and analysis of variance (ANOVA) were performed. To test for homogeneity between the groups, ANOVA was performed. To test the changes in time in EEG, two-way repeated measures ANOVA was performed. Lastly, a paired sample T-test was performed to compare outcome measures before and after the intervention for oral and mental health, and repeated measures ANOVA was performed to test differences between the groups.

## 3. Results

### 3.1. Participants’ General Characteristics

The three groups did not differ from one another at a statistically significant level in the demographic variables, except for the MMSE-K. In all groups, the proportion of females was greater when compared to the proportion of males, and the proportion of the age group of 75–84 was greater than any other age group. Regarding education level, high school graduation was the most common, and regarding socioeconomic status, middle class was the most common (Table 1).

### 3.2. Changes in EEG

The analysis of the EEG measurements at three different time points showed that the AL and PT differed significantly (*p* < 0.01 and *p* < 0.05 respectively). The post hoc test revealed that the AL significantly improved after the intervention in the CN (*p* < 0.01) and MCI group (*p* < 0.001) compared to pre-intervention. Post-intervention PT values were significantly improved than those measured after the first session (*p* < 0.05) in the CN group (*p* < 0.05) and those measured at pre-intervention in the MCI group (*p* < 0.05). There were no significant MD differences in the post hoc test (Table 2).

### 3.3. Changes in CBF

Four participants in the CN group were randomly selected for CBF measurements conducted a total of three times: prior to intervention, after the first session, and post-intervention. In one participant, CBF could not be measured because the cranium was too thick; thus, only the data from three participants were obtained. Measurements for each participant are shown in Table 3. CBF values recorded in the PCA were increased across the three measurement times in all three participants.

### 3.4. Effects of the Integrative Program on Oral and Mental Health

The mean O’Leary index score (dental plaque formation) decreased by 0.42 and 0.40 points in the CN and MCI groups, respectively (*p* < 0.001), and the Löe & Silness index score (gingivitis) decreased by 0.47 and 0.48 in the CN and MCI groups, respectively (*p* < 0.01). Saliva flow rate increased by 0.13 g/min in the CN group and by 0.15 g/min in the MCI group (*p* < 0.05). Additionally, both the O’Leary and Löe & Silness indices showed significant differences across the measurement times (*p* < 0.01) and in group × measurement time interaction (*p* < 0.05). The saliva flow rate showed a significant difference across the measurement times (*p* < 0.01).

In regard to mental health, a statistically significant difference was found in happiness in old age. Specifically, the happiness score increased post-intervention, by 6.94 and 7.30 points in the CN and MCI groups, respectively (*p* < 0.05), and the score was significantly different across the measurement times (*p* < 0.01) (Table 4).

## 4. Discussion

The current study was conducted to evaluate the effects of the integrative cognitive function improvement program on cognitive function and oral and mental health in cognitively normal and mildly impaired elderly people.

The prefrontal lobe is the area of the cerebral cortex most affected by aging, and cognitive function, which is strongly linked to the prefrontal lobe, can rapidly deteriorate due to aging [26]. In this study, EEG measurements in the prefrontal lobe that were indicative of AL and PT improved in the CN and MCI groups, and the improvement was statistically significant. Increased AL clears the head and improves immunity and attention, thus positively influencing cognitive function in elders. Typically, a higher stress level is associated with greater physical and mental fatigue, which lowers disease resistance and immunity. High physical stress refers to a poor physical condition due to tension and sleep problems, and high mental stress refers to the presence of anxiety, nervousness, and emotional instability [35]. In this study, physical stress was distinctly improved but mental stress was not, although the score increased. Thus, it is speculated that positive change may occur if intervention is provided for a longer term. 

Mean blood flow velocity is least influenced by physical changes and best reflects CBF status [27]. It is a widely known fact that average CBF velocity generally decreases with increasing age. In most elders, CBF is reduced if circulatory function decreases due to aging; hence, brain function is decreased in elder people with poor circulation and low CBF, resulting in cognitive deterioration [36]. Accordingly, to understand ways to lower the risk of cognitive deterioration, this study investigated the impact of the six-week-long integrative cognitive function improvement program on CBF velocity and found an increase in PCA alone. A recent study which demonstrated changes in CBF following participation in a cognitive and exercise program reported that CBF was increased post-intervention [27]. Hence, the integrative cognitive function improvement program seems to delay aging in the brain and the associated aging-induced decrease in CBF, thus helping in the functions of memory and learning in elders. To gather more evidence of this, future research interventions should be provided to a greater number of study participants.

Oral health is reported to greatly influence quality of life in old age [5]. In the elderly, mastication disorder, speech impairment, and pain in the mouth affect physical health, and if the problems affect interpersonal relationships and one’s social life, depression (a mental health problem due to social isolation) can develop [5,6]. Depression in old age is identified as a causative factor for dementia (a cognitive disorder) [8]. In the study, the analysis on oral health outcome measures showed that in the CN and MCI groups, the O’Leary and Löe & Silness indices significantly decreased, and the salivary flow rate significantly increased. This finding is consistent with the findings of a previous study, which examined the effects of oral health education utilizing a workbook and a mobile app [22]. It is speculated that in the current study, the use of a workbook after oral health education helped participants experiencing short-term memory problem easily understand the materials, as they could recall the lessons learned during the session. Additionally, the workbook had a positive effect in improving cognitive function. However, there were no statistically significant differences observed in tongue coating and oral muscle strength, though the measurement values trended in a positive direction. It is possible that a larger sample size and longer study period will be needed in the future in order to observe a statistically significant difference.

Depression is the most common mental health issue causing personal and social problems in old age [10]. Prevention of depression and the provision of appropriate treatment are challenging because most elders do not demonstrate an obviously depressive state. Depression is more likely to be manifested in physical symptoms. In elders whose cognitive function continues to deteriorate and in those with mild cognitive impairment, neurochemical changes due to depression induce dysfunction of the hippocampus and the anterior cingulate cortex, worsening a wide array of cognitive functions like memory, attention, inhibition, working memory, and visuospatial perception [9]. It is speculated that satisfaction with life improved in this study, as study participants experienced increases in interpersonal interactions and psychological support through cognitive, emotional, and physical activities over six weeks and that these factors contributed to enhanced happiness in old age. Therefore, it seems that the program is valuable as an intervention to promote mental health in elders whose cognitive function may be compromised.

Based on the study findings, the following suggestions for future studies and policy have been made in order to develop further specialized integrative programs for cognitive function improvement and actively treat the elder population. 

First, the current program showed equivalent outcomes in both the CN and MCI groups. However, responses to a program for elders may differ depending on the participants’ cognitive and health status. Accordingly, when an integrative program is designed and applied in the field, the status of target elders should be accurately identified and reflected in the program contents and operation. Second, the integrative program developed in the study could be utilized in a variety of settings, such as convalescent facilities and dementia prevention centers. Hence, local autonomous and central governments should establish funding and support systems for integrative programs to be aggressively utilized. Third, no previous studies, inside or outside of Korea, have ever conducted an experiment to test the effects of an integrative cognitive function improvement program by performing EEG and CBF studies; thus, the current study findings cannot be directly compared to any previous studies. Additionally, the study findings should be interpreted with caution because the level of cognitive function was not considered in selecting the control group. Further limitations are the small sample size and the inability to investigate related variables such as oral health status and oral care behavior of the participants. In future studies, positive intervention effects should be replicated by expanding the sample size and performing longitudinal research to investigate the long-term effects of integrative intervention programs.

## 5. Conclusions

In this study, cognitive, emotional, and physical activities were combined into an integrative cognitive function improvement program for the elder participants. Our findings showed that the program was found to be effective in improving AL and PT, increasing CBF in the PCA, reducing the extent of tongue coating and gingivitis, increasing saliva production, and enhancing happiness in old age in both the CN and MCI groups. It is hoped that integrative cognitive function improvement programs will be widely utilized to prevent the deterioration of oral and mental health and thereby improve the quality of life in the elder population.

## Figures and Tables

**Table 1:** General characteristics of the participants.

Variables		CN (*n* = 18)	MCI (*n* = 17)	Control Group (*n* = 17)	*p*-Value *
Sex	Male	3 (16.7)	2 (11.8)	1 (5.9)	0.861 ^b^
	Female	15 (83.3)	15 (88.2)	16 (94.1)	
Age, years	65–74	5 (27.8)	5 (29.4)	5 (29.4)	0.706 ^b^
	75–84	11 (61.1)	7 (41.2)	9 (52.9)	
	≥85	2 (11.1)	5 (29.4)	3 (17.6)	
Education	Primary school	5 (27.8)	3 (17.6)	7 (41.2)	0.518 ^a^
	Middle school	6 (33.3)	7 (41.2)	3 (17.6)	
	≥High school	7 (38.9)	7 (41.2)	7 (41.2)	
Economic level	High	5 (27.8)	3 (17.6)	6 (35.3)	0.842 ^b^
	Middle	8 (44.4)	8 (47.1)	7 (41.2)	
	Low	5 (27.8)	6 (35.3)	4 (23.5)	
Spouse	Widowed	9 (50.5)	8 (47.1)	6 (35.3)	0.877 ^b^
	Cohabitating	7 (38.9)	6 (35.3)	7 (41.2)	
	Separated or divorced	2 (11.1)	3 (17.6)	4 (23.5)	
MMSE-K		28.83 ± 0.38	20.11 ± 1.36	28.29 ± 3.17	0.001 ^c^
ADL		10.16 ± 0.70	11.11 ± 2.31	10.70 ± 1.31	0.211 ^c^
OHIP-14		56.27 ± 9.56	52.94 ± 17.17	52.35 ± 13.83	0.663 ^c^
GOHAI		42.05 ± 5.94	40.23 ± 8.23	38.64 ± 7.24	0.380 ^c^

Abbreviations: ADL, activities of daily living; CN, cognitively normal; GOHAI, geriatric oral health assessment index; MCI, mild cognitive impairment; MMSE-K, the Korean version of mini-mental status examination; OHIP-14, oral health impact profile. Data are presented as Mean ± SD for continuous variables and as *n* (%) for categorical variables. * *p*-values obtained from the ^a^ chi-square test, ^b^ Fisher’s exact test, and ^c^ one-way analysis of variance.

**Table 2:** Changes in electroencephalogram measurements.

Variables		CN (*n* = 18)	MCI (*n* = 17)	Control Group (*n* = 17)	Source	F	*p*-Value *
Arousal Level	Pre	5.46 ± 2.82	6.53 ± 2.39	5.10 ± 2.35	Group	1.134	0.330
	After one	4.83 ± 3.07	5.43 ± 3.20	4.30 ± 1.18	Time	10.213	0.001
	Post	3.49 ± 1.21 ^a^	3.68 ± 1.33 ^b,c^	4.11 ± 2.31	G × T	0.929	0.450
Physical Tension	Pre	32.98 ± 20.37	43.25 ± 24.02	34.67 ± 21.00	Group	0.880	0.421
	After one	34.65 ± 13.42	35.22 ± 10.04	32.80 ± 8.10	Time	3.977	0.025
	Post	24.60 ± 14.94 ^c^	26.77 ± 17.24 ^d^	31.11 ± 15.02	G × T	0.870	0.485
Mental Distraction	Pre	2.25 ± 1.95	2.32 ± 0.98	2.48 ± 1.43	Group	1.500	0.233
	After one	2.17 ± 0.89	2.31 ± 0.66	2.37 ± 0.61	Time	0.999	0.376
	Post	1.88 ± 0.81	2.03 ± 0.75	2.36 ± 0.94	G × T	0.198	0.939

Data are presented as mean ± SD. * *p*-values are calculated by two-way repeated measures ANOVA, GxT: GroupxTime. ^a–d^ The superscripts refer to the results of post hoc tests to test the differences between measurements at three time points. ^a^ Significantly different from pre-intervention (*p* < 0.01). ^b^ Significantly different from pre-intervention (*p* < 0.001). ^c^ Significantly different from after one session (*p* < 0.05). ^d^ Significantly different from pre-intervention (*p* < 0.05).

**Table 3:** Cerebral blood flow measurements of three participants in CN group.

Variables		Pre	After One	Post
Participant A	MCA	66.50	60.75	65.00
	ACA	39.75	44.00	35.50
	PCA	36.00	38.25	38.25
	VA	35.00	34.00	39.00
	BA	43.00	47.00	41.00
Participant B	MCA	54.75	56.00	58.50
	ACA	25.75	30.00	28.00
	PCA	26.75	28.00	29.25
	VA	28.00	28.50	27.50
	BA	20.00	22.00	29.00
Participant C	MCA	60.50	56.25	59.25
	ACA	32.50	34.00	29.75
	PCA	26.50	26.75	29.00
	VA	21.50	29.00	27.50
	BA	48.00	48.00	49.00

Abbreviations: ACA, anterior cerebral artery; BA, basilar artery; MCA, middle cerebral artery; PCA, posterior cerebral artery; VA, vertebral artery.

**Table 4:** Effects of the integrative program on oral and mental health.

Variables		Pre-Intervention	Post-Intervention	*p*-Value *	Source	*p*-Value **
O’Leary index	CN	0.86 ± 0.31	0.44 ± 0.23	0.001	Group	0.494
	MCI	0.92 ± 0.36	0.52 ± 0.25	0.001	Time	0.001
	Control	0.68 ± 0.27	0.58 ± 0.25	0.361	G × T	0.024
Löe & Silness index	CN	1.18 ± 0.36	0.71 ± 0.35	0.001	Group	0.400
	MCI	1.30 ± 0.48	0.82 ± 0.33	0.004	Time	0.001
	Control	0.97 ± 0.30	0.92 ± 0.35	0.643	G × T	0.010
Tongue coating	CN	3.11 ± 1.96	2.05 ± 2.01	0.111	Group	0.058
	MCI	3.29 ± 1.86	2.29 ± 1.64	0.125	Time	0.058
	Control	3.76 ± 1.98	3.58 ± 2.20	0.814	G × T	0.582
Saliva flow rate (g/min)	CN	0.25 ± 0.24	0.38 ± 0.27	0.023	Group	0.253
	MCI	0.21 ± 0.10	0.36 ± 0.19	0.006	Time	0.003
	Control	0.22 ± 0.16	0.22 ± 0.15	0.967	G × T	0.109
Anterior tongue strength(kPa)	CN	44.77 ± 10.09	46.16 ± 11.99	0.687	Group	0.290
	MCI	38.11 ± 13.42	42.58 ± 9.59	0.193	Time	0.249
	Control	37.29 ± 11.61	38.00 ± 7.72	0.819	G × T	0.689
Posterior tongue strength(kPa)	CN	40.72 ± 16.09	44.72 ± 13.86	0.219	Group	0.341
	MCI	36.41 ± 14.68	41.58 ± 11.09	0.085	Time	0.251
	Control	39.23 ± 5.82	35.82 ± 8.27	0.205	G × T	0.084
Cheek strength (kPa)	CN	25.61 ± 10.05	27.72 ± 9.34	0.490	Group	0.758
	MCI	24.41 ± 7.96	26.35 ± 13.27	0.596	Time	0.437
	Control	24.47 ± 6.02	25.00 ± 14.12	0.883	G × T	0.936
Mental health	CN	64.61 ± 13.17	67.72 ± 6.53	0.414	Group	0.629
	MCI	61.64 ± 9.23	66.11 ± 11.65	0.176	Time	0.072
	Control	63.52 ± 6.60	66.00 ± 8.59	0.310	G × T	0.902
Happiness	CN	61.83 ± 11.24	68.77 ± 9.86	0.036	Group	0.546
	MCI	62.11 ± 12.99	69.41 ± 11.29	0.045	Time	0.018
	Control	62.88 ± 10.79	62.52 ± 9.88	0.919	G × T	0.190
Social support	CN	62.72 ± 12.52	65.72 ± 7.61	0.430	Group	0.547
	MCI	57.82 ± 21.65	62.17 ± 16.93	0.372	Time	0.212
	Control	62.29 ± 12.93	63.76 ± 10.20	0.685	G × T	0.882

Data are presented as mean ± SD. * Difference between pre- and post-intervention was calculated using a paired sample *t* test. ** Repeated measures ANOVA, GxT: GroupxTime.

## Data Availability

The data that support the findings of this study are available from the corresponding author upon reasonable request.

## Supplementary Materials

The following supporting information can be downloaded at: https://www.mdpi.com/article/10.3390/ijerph192114339/s1, Table S1: Integrative cognitive function improvement program; Table S2: Details of general characteristics measurements; Table S3: Details of oral health status measurements; Table S4: Details of mental health status measurements; Table S5: Summary of outcome measures.
