# Supplementary material for: Effects of Integrative Cognitive Function Improvement Program on Cognitive Function, Oral Health, and Mental Health in Older People: A Randomized Clinical Trial
Source: Int J Environ Res Public Health. 2022 Nov 2;19(21):14339. doi: 10.3390/ijerph192114339 (PMC9659034; doi:10.3390/ijerph192114339)
Supplement: Supplementary file 1 [file ijerph-19-14339-s001.zip › ijerph-1921037-supplementary.pdf]

Supplement for "Effects of Integrative Cognitive Function Improvement Program on Cognitive Function, Oral Health, and Mental Health in Older People: A Randomized Clinical Trial"

Eun-Seo Jung[1], Yoon-Young Choi[2], Kyeong-Hee Lee[1]

[1]Department of Dental Hygiene, College of Bioecological Health, Shinhan University

[2]Private practice, Nohyeong 14-gil, Jeju 63083, Republic of Korea.

Supplementary Table S1 Integrative cognitive function improvement program

Program		Contents	Time(min)
Cognitive activities	Oral health education	1. Oral health problems in older adulthood<br>2. Oral care	15
	Workbook	Line drawing, coloring, puzzles, filling the blanks, etc.	15
Emotional activities	Music	Listening to music, singing (pop songs, children's songs), rhythmic movements	15
	Laugh	Greeting laughter, laughter in daily live, laughter exercise, clapping and laughing, body laughter, singing laughter	15
Physical activities	Whole-body exercise	1. Whole body warm-up<br>2. Shoulder muscle exercise<br>3. Flexor muscle stretching<br>4. Cool-down	20
	Oral exercise	1. Oral exercise that promotes saliva production<br>2. Oral exercise to strengthen mastication<br>3. Oral exercise to strengthen swallowing<br>4. Oral exercise to strengthen talking	10

Supplementary Table S2 Details of general characteristics measurements

MMSE-K

The MMSE-K consisted of a total of seven domains: orientation to time (five points), orientation to place (five), registration (three), attention and calculation (five), recall (three), language (eight), and visual construction (one). By adding up the domain-specific scores, the final score ranged between 0-30, and the higher the total score, the better the cognitive function.

ADL

The ADL consisted of a total of 10 items regarding eating, bathing, grooming, bowel control, bladder control, dressing, toilet use, making a bed, walking, and using the stairs. Each activity was ranked on a three-point scale (one point for completely independent, two points for partially independent, and three points for completely dependent). The total score ranged from 10-30, and the lower the score, the better the performance in ADL.

OHIP-14 and GOHAI

The OHIP-14 consists of 14 items and the GOHAI 12 items, and all items were ranked on a five-point Likert scale. The highest possible score was 70 and 60 points in OHIP-14 and GOHAI, respectively. With both instruments, the higher the score, the lower the oral health-related quality of life.

Supplementary Table S3 Details of oral health status measurements

O'Leary index

The O'Leary index is a tool quantifying the status of oral hygiene. In this study, to examine dental plaque formation, a plaque disclosing agent was applied to four tooth surfaces (mesial, distal, buccal, and lingual); a score of zero indicated dental plaque was not visually observed, and score of one indicated dental plaque was visually observed. Because elder patients often present with partial edentulism, in the study, all teeth were assessed for the O'Leary index. The index was determined by dividing the total dental plaque score by the number of examined tooth surfaces, and lower O'Leary index values indicated better dental plaque management.

Löe & Silness index

To quantify the severity of gingival inflammation with the Löe & Silness index (a commonly used measure for gingivitis), four surfaces (mesial, distal, buccal, and lingual) of each tooth were scored, with a score of zero indicating no inflammation, a score of one mild inflammation, a score of two moderate inflammation, and a score of three severe inflammation. All teeth were scored for this index, as well, because of partial edentulism in the elder participants. The Löe & Silness index was computed by dividing the total gingivitis score by the number of examined tooth surfaces. The lower the index value, the healthier the gingiva.

Tongue coating

To assess the extent of tongue coating, the tongue was divided into a total of six segments. Each segment was scored, with a score of zero indicating no coating and a score of one indicating mild coating. The total score ranged from zero to six points, with a higher total score indicating more severe coating of the tongue.

Saliva flow rate

To assess salivary flow rate (unstimulated saliva production), participants were instructed to swallow the saliva present in the mouth and then collect fresh saliva in a paper cup for five minutes. The total amount of collected was divided by 5 and written as g/min. Because the goal of this study was not to diagnose oral dryness but to examine changes in saliva production pre- vs. post-intervention, weight (g), not volume (ml), was used as the unit of saliva production. A higher weight indicated a greater amount of unstimulated saliva production.

Oral muscle strength

To measure pressure in the anterior tongue, the research assistant placed the bulb so that the edentulous alveolar ridge of the hard palate and the anterior 10 mm of the tongue would touch. To measure pressure in the posterior tongue, the bulb was placed such that the posterior hard palate and the tongue would touch. With the bulb in place, participants were instructed to press it with the tongue as hard as possible for two seconds. To measure cheek muscle strength, the bulb was placed between the teeth and the buccal vestibule, and participants were instructed to lightly close the mouth and press the bulb as hard as they could for two seconds. The higher the pressure level, the stronger the oral muscles.

Supplementary Table S4 Details of mental health status measurements

**KGHQ**	
	The KGHQ consisted of a total of 20 five-point Likert scale items. The highest possible total score was 100 points, and the higher the score, the better the mental health.
**Happiness in old age**	
	The scale consisted of a total of 19 five-point Likert scale items; the highest possible total score was 95 points, with higher scores indicating greater happiness.
**MOSSSS**	
	MOSSSS consisted of a total of 19 five-point Likert scale items. The maximum total score was 95 points. The higher the score, the greater the level of social support.

Supplementary Table S5 Summary of outcome measures

Measurement items	Details	Research instruments/equipment
Cognitive function	Electroencephalogram	Neurobrain (Neuro21, Korea)
	Cerebral blood flow	Transcranial Doppler ultrasound(TCD)
Oral health	Dental plaque	O’Leary index
	Gingivitis	Löe & Silness index
	Tongue coating	Winkel's Tongue Coating (WTC)
	Saliva flow rate	Unstimulated saliva production (g)
	Oral muscle strength	Iowa Oral Performance Instrument (IOPI)
Mental health	Mental health in old age	Korean General Health Questionnaire (KGHQ)
	Happiness in old age	Subjective Happiness Scale
	Social support	The Medical Outcomes Study Social Support Survey (MOSSSS)
